# Stakeholder Perspectives on Advancing Understanding of Prenatal Opioid Exposure and Brain Development From the iOPEN Consortium of the Healthy Brain and Child Development Study

**DOI:** 10.3389/fpsyg.2021.698766

**Published:** 2021-07-30

**Authors:** Jennifer L. DiPietro, Kristen L. Mackiewicz Seghete, Elizabeth E. Krans, Kasey Edwards Snider, Reina Bower, Kea Parker, Janie Gullickson, Alexandra S. Potter, Hugh Garavan, Tessa C. Vatalaro, Moriah E. Thomason, Elinor L. Sullivan, Alice M. Graham

**Affiliations:** ^1^Department of Psychiatry, Oregon Health and Science University, Portland, OR, United States; ^2^Department of Obstetrics, Gynecology, and Reproductive Sciences, University of Pittsburgh, Pittsburg, PA, United States; ^3^Magee-Women's Research Institute and Foundation, Pittsburgh, PA, United States; ^4^Project Nurture Providence, Portland, OR, United States; ^5^Mental Health Addiction Association of Oregon, Portland, OR, United States; ^6^Department of Family Medicine, Oregon Health and Science University, Portland, OR, United States; ^7^Department of Psychiatry, University of Vermont, Burlington, VT, United States; ^8^Department of Child and Adolescent Psychiatry, New York University Medical Center, New York, NY, United States; ^9^Department of Population Health, New York University Medical Center, New York, NY, United States; ^10^Department of Behavioral Neuroscience, Oregon Health and Science University, Portland, OR, United States

**Keywords:** stakeholders, patient advocates, opioids, addiction, pregnancy, *in utero* exposure, neurodevelopment, Healthy Brain and Child Development Study

## Abstract

**Introduction:** There is a dire need for research regarding the implications of opioid use during pregnancy on fetal and childhood development to better inform both medical practice and policy. The Healthy Brain and Child Development Study will examine brain and behavioral development from birth through the first decade of life. Due to large scope and anticipated complexity of this initiative, an 18-month planning phase was implemented across 28 sites across the nation. A core element of the Phase I initiative involved the development of Stakeholder Advisory Committees to inform the next phase of the initiative.

**Methods:** Phase I stakeholder meetings were conducted at Oregon Health and Science University, New York University Langone Medical Center, the University of Pittsburgh, and the University of Vermont to better understand perspectives and inform upcoming research. Despite differences in the structure of the stakeholder meetings by site, the overarching goals for the meetings included establishing relationships, gathering input, and learning about research engagement. Documents from each meeting were reviewed for location, duration, attendees, common research themes, and pertinent suggestions for improving research approaches.

**Results:** All stakeholders had high levels of interest in research for pregnant people with substance use disorders and agreed on research priorities including collaboration, connection, communication, and support. Different stakeholders offered unique perspectives on various aspects of study design and themes that emerged through meetings.

**Discussion:** Overall, there was excitement about the research, especially the opportunity to include the voices of people with lived experience; collaboration between providers, peer support specialists, patients, and others; and excitement around contributing to research that could elucidate new and pertinent findings in the realm of addiction medicine and child development. Sites also found that there is mistrust between people with substance use disorder and the medical system, and this could be addressed by including people with lived experience on the research team, forming connections, communicating clearly, training the research team in implicit bias, and practicing trauma-informed care. In conclusion, these stakeholder meetings provided valuable information for structuring upcoming studies; however, researchers would have benefitted from more time and more opportunities for in-person connection.

## Introduction

The increased prevalence of opioid use during pregnancy and the subsequent potential effects of *in utero* opioid exposure on children represent a dual maternal-child health epidemic in the United States. Between 1999 and 2014 the prevalence of perinatal opioid use disorder (OUD) increased from 1.5 to 6.5 per 1,000 deliveries (Goodman et al., 2020). The number of infants diagnosed with neonatal abstinence syndrome (NAS) grew ~5-fold (Krans and Patrick, 2016), currently accounting for about 50% of all NICU hospital days in some communities and $1.5 billion in hospital charges (Tolia et al., 2015; Patrick et al., 2017). These numbers demonstrate a dire need for research regarding the immediate and long-term implications of opioid use during pregnancy on maternal outcomes and fetal and childhood development to better inform both medical practice and policy.

The National Institute of Drug Abuse, in partnership with multiple other NIH institutes, has issued a request for proposals to conduct the Healthy Brain and Child Development (HBCD) Study, a groundbreaking project that would examine brain and behavioral development from birth through the first decade of life (Volkow et al., 2021). The study will establish a national consortium of ~25 research sites across the country and will involve 7,500 children and their families, oversampling for infants who were exposed to opioids *in utero*. This study has the potential to substantially impact scientific understanding of early brain development and mental health in the context of environmental influences beginning *in utero*, and even prior to conception. Importantly, there is an emphasis on capturing a wide range of domains and exposures, with understanding that many of the circumstances that accompany substance use, such as poor maternal nutrition and unstable housing, also have the potential to influence neurodevelopment. Co-occurring factors such as maternal trauma and psychiatric history, experiences of discrimination, and variation in socioeconomic conditions will be investigated as well.

An important consideration in an undertaking such as this is how to effectively engage and support people using substances during pregnancy in longitudinal research, many of whom have historically and traditionally been underserved and stigmatized in multiple medical, support, and research domains. There is a growing awareness in the field of intervention research of the need for a paradigm shift away from academic, top-down, clinical trials toward the development of interventions informed by patients, providers, and real-world implementation settings (Schindler et al., 2017). Exclusion, or limited inclusion, in research of patients or people with lived experience of substance use during pregnancy marginalizes their voices in academia (i.e., research), science and health policy. The current opioid epidemic has highlighted the negative impacts of this pervading paradigm on pregnant people who use substances and their infants as evidenced by the persistence of punitive vs. treatment-oriented policies across many states (Krans and Patrick, 2016). Existing data also indicates that gaining patient perspectives can increase engagement in both research and clinical care (Brett et al., 2014). Thus, gaining input from people with lived experience using substances during pregnancy (current or past) has significant potential to improve research in the realms of study design, innovation, recruitment and retention, ethical standards, and real-world translation potential.

Due to the large scope and anticipated complexity of the HBCD initiative, an 18-month planning phase, known as HBCD Phase I, was implemented across 28 sites across the nation. Sites were tasked with both preparing local infrastructure and piloting activities anticipated to feature centrally in Phase II. A core element of the Phase I initiative involved the development of Stakeholder Advisory Committees to inform the next phase of the HBCD initiative. Key to the Stakeholder Advisory Committees was involvement of people with lived experience of using substances during pregnancy and care providers. The overarching goals of these committees were to: (1) obtain input regarding the best ways to ethically and sustainably conduct research with pregnant people, parents, infants and children impacted by substance use and other sources of adversity; (2) learn what engagement, partnership and collaboration with researchers means to different communities and organizations; (3) examine research attitudes and priorities among different communities. Research guided by community insights and perspectives is more likely to translate into meaningful interventions going forward.

## Materials and Methods

### iOPEN Sites

As part of Phase I of the HBCD initiative, one of the consortiums established was the Investigation of Opioid Exposure and Neurodevelopment (iOPEN). The iOPEN consortium consisted of a set of linked sites that participated in HBCD Phase I, including Oregon Health and Science University, New York University Langone Medical Center, the University of Pittsburgh, and the University of Vermont. Stakeholder meetings were conducted at these iOPEN sites from 2019 to 2020 to better understand stakeholder perspectives and inform upcoming research. The iOPEN Phase I study was approved by the Institutional Review Board (IRB) at New York University.

### Stakeholders

In the context of this project, the following people were considered stakeholders: (1) people with lived experiences of substance use during pregnancy, including opioid use; (2) medical providers or other care providers for pregnant people using substances and their infants; and (3) people making decisions at the individual or policy level with direct impacts on pregnant people who use substances and their infants. See Table 1 for a detailed description of stakeholders involved at each site.

**Table 1:** Composition of stakeholder meetings by site.

**Site**	**Stakeholders Represented**
Oregon Heath and Science University (OHSU)	People with lived experience (2), peer support specialists (2), people affiliated with an OUD treatment program specializing in pregnancy (3), people affiliated with local non-profits (2), state/local health authority representatives (2), family medicine physicians (2), an OB/GYN (1), nurse-midwife (1), doula (1), developmental psychologist (1), child and adolescent psychologist (1), neuroscience researcher (1), and OB research associate (1)
University of Pittsburgh Medical Center (UPMC)	Mother with lived experience (1), RN (1), physician researcher (1), OUD treatment provider specializing in the care of pregnant and parenting persons (1), PhD investigator (1), and research coordinator (1)
New York University Langone Medical Center (NYU)	People affiliated with OUD treatment program specializing in pregnancy (three agencies), child welfare representatives (6), addiction medicine physicians (2), and a young mother with lived experience (1)
University of Vermont (UVM)	Person affiliated with OUD treatment program specializing in pregnancy (1), child welfare representative (1)

### Stakeholder Meetings

#### Meeting Goals

Despite differences in the structure of these meetings by site, the overarching goals for all sites were as follows:

1. Establish a dialogue and build relationships with key organizations and stakeholders to support the ultimate goal of conducting a large-scale study of early brain development with families facing multiple sources of adversity, and particularly experiences of substance use during pregnancy.
2. Get input regarding the best ways to ethically and sustainably conduct research with pregnant people, parents, infants and children impacted by substance use and sources of adversity.
3. Learn about what engagement, partnership, and collaboration means to different stakeholders.
4. Learn about stakeholder attitudes toward research and the medical community more broadly.

#### Individual Sites

##### Oregon Health and Science University

Two group meetings each lasting 60 min were structured with a list of questions designed to elicit information and discussion about research attitudes and priorities. Prior to the second meeting, a survey and email were sent to all attendees of the first meeting to gather input on the topics and potential additional attendees for the second meeting. Notes were taken during meetings to capture elements of discussion. Participants were also invited to submit written responses to questions to increase inclusiveness of preferred communication style. The first meeting included 14 attendees and was in-person, while the second meeting included eight attendees and was virtual.

##### University of Pittsburgh Medical Center

One in-person group meeting lasting 60 min was conducted in a free discussion format with six attendees including treatment providers and a peer navigator from the Pregnancy and Women's Recovery Center, an outpatient OUD treatment program for pregnant and parenting women.

##### New York University

Twelve meetings, each ~60 min in duration, were held with community organizations and medical centers including the Odyssey House (a residential treatment facility for pregnant mothers with OUD), Administration for Children's Services (Department for Child Welfare), Cooper University Healthcare (outpatient OUD treatment program), and Montefiore Medical Center (hospital OUD treatment program for mothers). Four of these meetings were held in-person, and the other eight were virtual.

##### University of Vermont

Two 30-min, in-person one-on-one meetings were held in a free discussion format. The first meeting included a child welfare representative, and the second meeting included a person who was affiliated with a treatment program specializing in pregnancy.

### Analysis of Meeting Notes

Documentation from each site's meeting(s) in the form of a template were reviewed (JD) for location, duration, and attendees. See Appendix A for the template. After gathering basic details on the meeting formats and attendees, free form notes were further reviewed to identify common research themes and pertinent suggestions for improving research approaches. Common themes across the sites and implications for future research were identified and summarized.

## Results

Across all four sites, researchers were asked to record themes that emerged from meetings. All identified themes have been summarized and divided by category below. Themes are intended to inform planning and development of Phase II of the HBCD study. Implementation (e.g., practical, ethical) and measurement of outcomes would be a part of the evolving Phase II process, with site-specific considerations (e.g., geographic location, demographics).

### Strong Interest in Research

All stakeholders had high levels of interest in the proposed research, and there is a desire across sites to work collaboratively with existing systems of care for pregnant people with substance use disorders (SUD). At the OHSU site there was excitement about the potential for research to address questions about the effects of substance use during pregnancy on child development as well as the opportunity for people with lived experience to have a voice in research. Involvement in research could increase connection between participants and bring a sense of meaningful contribution. People with lived experience and peer support specialists shared experiences of mistrust and frustration with prior research as these studies did not account for key potential confounding factors, such as socioeconomic status. Additionally, both people with lived experience and providers were frustrated about the lack of clear and consistent communication from providers and different agencies about what might be harmful to a developing fetus and the potential implications for child development.

### Research Priorities: Collaboration, Connection, Communication, and Support

Priorities discussed by the NYU site included a desire to work collaboratively in order to fund research and treatment initiatives, decrease undue family separation related to substance use, and effectively connect participants to research opportunities. OHSU stakeholders, specifically people with lived experience and peer support specialists, stated that their priorities were to: improve the design of future research studies so they can disentangle the effects of co-occurring factors, like prenatal stress, trauma history, and food security, from the potential effects of substance use during pregnancy; address the fear, guilt, and shame often experienced by parents who have used substances during pregnancy by initiating studies with larger sample sizes with the ability to better understand potential effects of substance use on offspring; address conflicting information provided to pregnant people using substances or in treatment during pregnancy by providing communications that are informed by the current evidence base; and study protective factors for parents and children instead of solely focusing on adverse outcomes. Providers at these meetings stated priorities such as decreasing fear and discrimination among medical providers through providing a more solid research base on pregnancy and SUD, and creating a structure for research projects that allows providers and policy makers to gain information, feel supported, and reduce bias against pregnant people using substances. Lastly, all stakeholder groups at the OHSU site meetings spoke to the importance of creating a structure for research projects that gives participants the opportunity to feel connected to others with lived experience and to the medical community, and to feel that they are making a valuable contribution—essentially using research participation as a way to decrease isolation and shame and also contribute to synthesizing current information and recommendations regarding effects of substance use during pregnancy and treatment options for patients and providers.

### Barriers and Challenges

Sites agreed that institutional barriers and the COVID-19 pandemic could pose challenges for research, along with limited funding opportunities and access to data. The UVM site stakeholders specifically mentioned the potential challenge of facilitation of consent for infant participation in the study if birth parents temporarily or permanently lose guardianship. Stakeholders at the UPMC site discussed concerns about how willing pregnant people might be to complete an MRI and logistical barriers that might make completing the MRI difficult, such as transportation to and from the MRI location and childcare during the MRI. They also made points about the use of language when discussing the research—for example, not implying that there is a problem with opioid use or participants' infant's brain or making people feel like they will be experimented on during this study. OHSU site stakeholders brought up a few challenges pertaining to participants having prior negative experiences with the medical system and research; additionally, participants could be concerned about the study results indicating negative impacts of OUD on child outcomes, which could deter participation.

### Achieving Research Priorities

General suggestions included: meeting patients “where they are” without any expectations; practicing trauma-informed care; demonstrating an understanding of the social determinants of health; forming relationships with study participants; providing remote support; frequent check-ins to gauge population needs; and understanding participants' motivations for participation. OHSU site stakeholders suggested implicit bias training so that researchers are cognizant of inherent bias that can exist at different points of the research process. NYU site stakeholders discussed data sharing and networking between providers and investigators in partnerships. The UVM site has a coordinated care group for all pregnant people who are in substance use treatment, and researchers at that site have been invited to participate in these meetings that include clients.

### Research Strategies

#### Inclusion and Exclusion Criteria

Stakeholders at the OHSU site discussed the importance of considering factors that co-occur with substance use in study design, data analysis, and contextualization of interpretation, including low socio-economic status, trauma, mental health disorders and symptoms, poor nutrition, and experiences of discrimination. NYU site stakeholders suggested expanding the scope of the study to pregnant people with all substance use disorders as opposed to focusing solely on OUD.

#### Recruitment Strategies

Sites agreed that including people who have lived experienced with substance use during pregnancy on the research team and community advisory board would be an important way to form personal connections with research participants and better communicate information about research, with the added potential of decreasing mistrust in the healthcare system. The UVM site planned to recruit participants by having team members present at coordinated care meetings that help plan for pregnancy for people in SUD treatment. NYU site stakeholders mentioned a preference for in-person recruitment at the facility, however, with COVID-19 restrictions that might not be possible for 2022. In lieu of in-person recruitment, participants could be recruited through hospital system medical record data. Lastly, the UPMC site discussed how providing pregnant people with the MRI imaging taken of their baby could be seen as recruitment incentive, but that teams should also consider incentives such as money, food, diapers, or transportation.

#### Retention Strategies

UPMC site stakeholders suggested obtaining multiple contacts from participants, such as family members and friends, and gaining permission to contact those people throughout the study. NYU site stakeholders spoke to the importance of feeling a partnership between the study participants and stakeholders, and potentially forming a partnership with housing authorities in the local jurisdiction as well. Similar to recruitment strategies, OHSU site stakeholders emphasized having people who have lived experience with substance use during pregnancy on the team to promote retention and engagement with the study. Additionally, it is important for researchers to understand the motivation behind participation—understanding their reasons for joining the study could make their participation more meaningful and promote retention.

#### Frequency of Study Visits

The UPMC site was the only site to raise discussion of the frequency of study visits; discussion was broad, with no specific visit timeline suggested. Stakeholders thought that telehealth/virtual visits would be most ideal for this study, especially if study visits were tied to treatment program visits.

#### Composition and/or Role of Community Advisory Board

Sites agreed that the community advisory board should include people with lived experience of substance use during pregnancy, peer support specialists, healthcare providers from different disciplines, policy makers, and child welfare representatives. This variety of different perspectives will be important for shaping research and also creates the opportunity to further communication between these groups. Multiple sites mentioned high levels of interest in supporting ongoing dissemination of findings with relevant service sectors and the community advisory board.

#### Key Ethical and Legal Considerations

Some important ethical considerations that emerged from the meetings included ensuring that participants would indeed benefit from the study, and that their experiences with the study would not cause further mistrust of the healthcare system. It will be important to consider the implications of parental rights in the event of guardianship changes that might result in retention of the parent or child in the study.

### Individual Stakeholder Contributions

Different stakeholders offered unique perspectives on different aspects of study design and themes that emerged through meetings. People with lived experience and peer support specialists offered firsthand experiences with difficulties navigating healthcare, including perspectives on judgment from providers, and general distrust of the system based on past trauma. They emphasized the importance of including people with lived experience on the research team to create a welcoming atmosphere and reinforce trust in the research mission. Healthcare providers spoke to the lack of information and knowledge about impacts of opioids on fetal brain development, and the need for concrete evidence to give patients during treatment. Child welfare representatives were able to highlight legal considerations regarding custody changes, while policy makers offered perspectives on how information gathered from future research could improve the quality of patient education and legislation.

**Figure 1:**
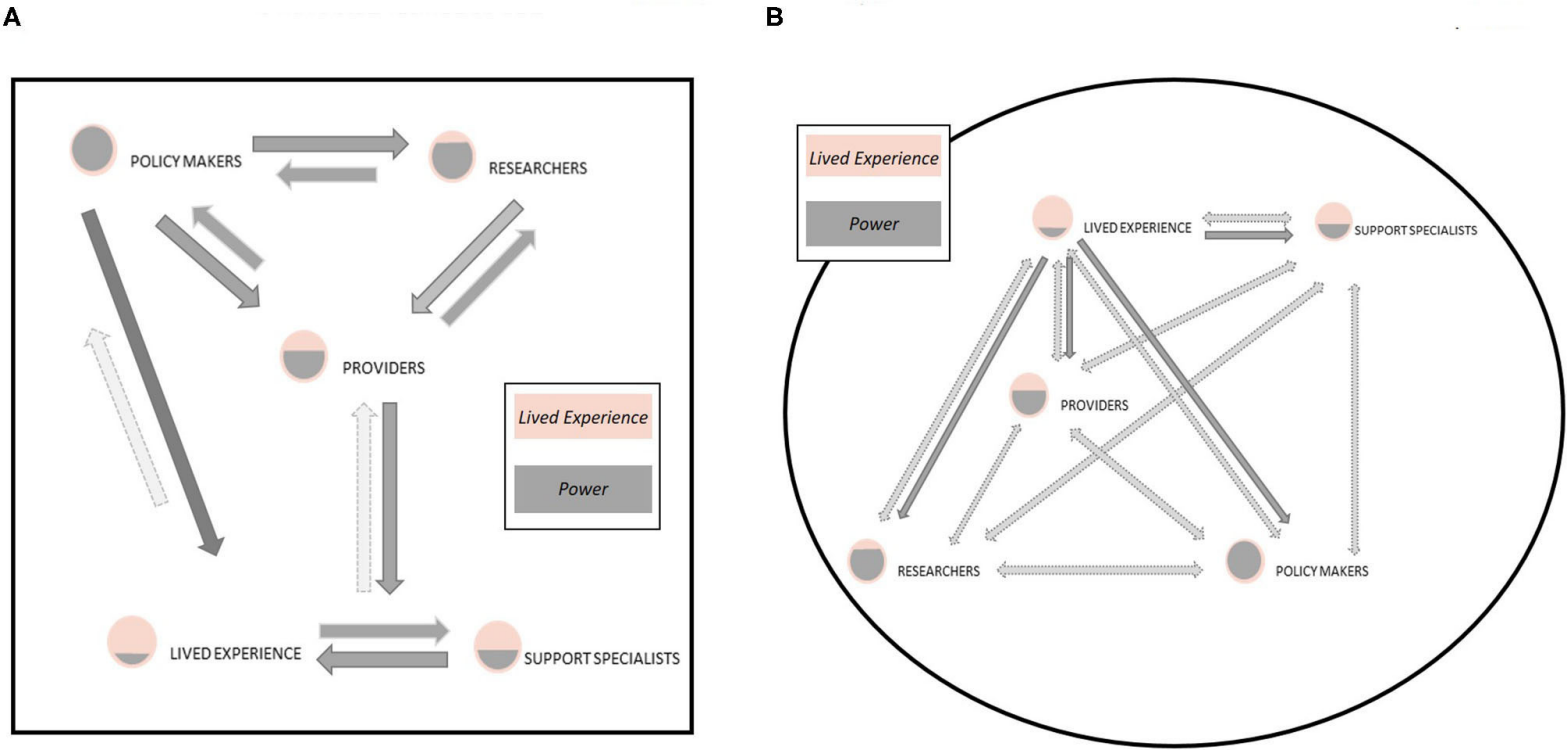
Model of **(A)** traditional sources of influence and contributors to research, often policy makers (including funding agencies) and researchers. This reflects a top-down, hierarchical model of power and privilege in the design, dissemination, and implementation of research outcomes. In this model, individuals with lived experience often have the least direct contribution to research yet are impacted the most by the direct products and dissemination (or lack therefore) of research. In contrast, we present a new model **(B)** that enhances the voice of individuals with lived experience in the research process and suggests a more bidirectional and inclusive model. Elevating the role of individuals with lived experience within the research process provides valuable insight to ensure the research process is inclusive, research aims are reflective of actual need, and research products address questions and weaknesses that the research community and policy makers have overlooked or ignored.

Figure 1 illustrates a conceptual framework informed by stakeholder meetings, which places patients and individuals with lived experience at the center, and demonstrates the concentric levels of contact between stakeholders including peer support specialists, healthcare providers, child welfare, and policy makers. In practicing patient-centered research, patients occupy the center space, with peer support specialists in immediate contact with them, as those who assist patients firsthand in navigating the healthcare system and advocating for their needs. Healthcare providers represent the next layer of the concentric model, as those who care for patients in the medical setting, both in prenatal and SUD capacities. Child welfare and policy makers represent the final layers of the model, as they have less direct contact with patients, but are important in making guardianship and custody decisions and crafting legislation that impacts pregnant people with SUD and their infants.

## Discussion

As HBCD focuses on understanding the brain and behavioral development of children exposed to opioids *in utero* and their parent's ability to effectively provide care for their child, it is essential that stakeholder input, especially from people with lived experience, inform the study design. In an effort to understand the patient voice in HBCD, stakeholder meetings were conducted across iOPEN consortium sites to aide in HBCD formation and design. Despite each site taking a different approach to the stakeholder meetings, common themes and implications emerged. Specifically, there was excitement about the research, especially the opportunity to include the voices of people with lived experience, and the ability to contribute to a broader evidence base pertinent to addiction medicine and child development in the context of *in utero* exposure to opioids. All types of stakeholders suggested research priorities should include collaboration between providers, peer support specialists, patients, and others.

There were also a significant number of strategy suggestions coming out of the stakeholder meetings. Sites found that there is mistrust between people with SUD and the medical system, and this is something that could be addressed by including people with lived experience on the research team, forming connections, communicating clearly, training the research team in implicit bias, and practicing trauma-informed care. There was a strong emphasis on the need for rigorous research designs that could effectively delineate the effects of factors that frequently co-occur with SUD during pregnancy from the effect of SUD on fetal, infant, and child development, such as low socioeconomic status or poor nutrition. Another unanimous priority was that providers and participants should all feel supported in providing/receiving care in the research structure, and have an understanding of the social determinants of health. Research teams should include people with lived experience to improve both recruitment and retention of participants, and researchers should understand what is motivating participants to join the study. There should be ample incentives for participation, such as money, food, or transportation. It would likely be most feasible to recruit from medical record data and conduct remote study visits when possible, due to restrictions secondary to the COVID-19 pandemic. Community advisory boards should include stakeholders from a wide array of backgrounds, including people with lived experience, peer support specialists, providers, policy makers, and child welfare organizations, to promote connectedness and collaboration, and bring different perspectives to the table.

Through conducting these stakeholder meetings, several challenges emerged, including the rapid timeline of grants limiting the time for relationship building, COVID-19 limiting in-person meetings and increasing daily challenges for providers, patients, and policy-makers, and academic/research-focused language in presenting and writing up the study. Multiple sites shared that in-person stakeholder meetings were more effective in fostering community and keeping people engaged, while virtual meetings were more accessible and could increase collaboration. These challenges posed important questions to consider for future stakeholder meetings. First, is there a best format for meeting or does it vary greatly depending on the site and on current conditions (for example, the COVID-19 pandemic)? Are group or one-on-one stakeholder meetings more effective for gathering input? Additionally, how can we more effectively involve stakeholders at every stage of the research process, from meeting planning through interpretation of results?

Figure 1 demonstrates a concentric model of the layers of contact and intersection between the stakeholders involved in this process. It is critical to have the patient perspective informing our research goals and strategies, and incorporation of stakeholders across the levels of the concentric model further strengthen recommendations and translatability of research. However, there is limited data on the patient experience of pregnant people with OUD, specifically how pregnant people with OUD perceive information provided by medical professionals about the effects of *in utero* drug exposure on their developing infants, during pregnancy, infancy, and into childhood. Existing data indicates a significant lack of trust, and many barriers to interacting with medical care providers for substance use treatment and by extension, researchers operating in medical settings (Goodman et al., 2020). Barriers include lack of insurance, high costs of care, long waiting lists to obtain care, and a lack of transportation (Goodman et al., 2020). During pregnancy, accessing treatment can be particularly difficult due to the stigma surrounding substance use during pregnancy and the threat of the legal system intervening through Child Protective Services (Goodman et al., 2020). Despite the condemnation of punitive treatment for drug use during pregnancy by national associations such as the American Academy of Pediatrics (Patrick et al., 2017), 18 states still classify substance use during pregnancy as criminal child abuse, which can result in termination of parental rights (Krans and Patrick, 2016). This represents a lost opportunity, as pregnancy is a turning point for many people during which they decide to seek help for substance use, both for the health of themselves and their infants, and engage with the healthcare system (Goodman et al., 2020).

Qualitative research has shown that pregnant people with OUD need to have access to “gender-specific, family-friendly addiction treatment programs, psychosocial services, and mental health treatment” due to high rates of trauma and abuse (Patrick et al., 2017). Unfortunately, trials of mental health interventions during pregnancy often exclude pregnant people using substances (Seghete et al., 2020), limiting the evidence base for selecting appropriate interventions addressing mental health in this population. Addressing logistical barriers also appears to be critical, as an important factor contributing to continuation of treatment is the availability of on-site childcare and services (Patrick et al., 2017). Those who attended substance use treatment program support groups cited their peers as “significant source(s) of support and information,” and many people found comfort in hearing the stories of other births following treatment for OUD (Goodman et al., 2020). One-to-one clinical support to assist patients in navigating the healthcare system and other sources of assistance has also been cited as a way to help people engage with medical providers, overcome barriers, and set goals for themselves and their newborns (Cochran et al., 2019).

Peer support specialists are the next level out in the concentric model, as they are closely associated with patients and focus specifically on supporting pregnant people with OUD in the process of seeking treatment and navigating the healthcare system. They are uniquely positioned to ensure that a patient's needs are being met and that their voices are heard. Peer support specialists are often also people with lived experience with substance use during pregnancy and parenting. Medical providers are the next level out from patients and peer support specialists, as they provide direct care to pregnant people with OUD and their children. In the research context, they provide unique perspectives on how research is interpreted, what information is shared with patients, and directly influences care for this population. They can additionally provide insights on barriers to research participation and factors that may facilitate research engagement. Lastly, they can give voice to what data and evidence is missing that might help them better care for pregnant patients with OUD.

Next, child welfare agencies are stakeholders in research regarding substance use during pregnancy since they theoretically rely on this research to determine safety of infants and families, and make critical decisions about child guardianship and custody. Child welfare agencies become involved with pregnant people in OUD treatment in states that require intervention, and often work with families both during and after treatment to assure that newborns are in safe home environments. Lastly, policy makers are those responsible for developing and implementing legislation that impacts pregnant people with OUD and their newborns. They, too, rely on research and research dissemination to inform legislation. Policies then impact the care given by healthcare providers and the extent to which child welfare agencies become involved during and after pregnancy.

As these stakeholder meetings were conducted at the iOPEN consortium sites as part of the Phase I initiative of the HBCD study, stakeholder meetings were limited in scope to support the aims of planning and development of Phase II of the HBCD study. Therefore, stakeholders with lived experience were most representative of individuals with lived experience of using opioids during pregnancy. Phase II of the HBCD study will provide an opportunity to expand stakeholder groups that will evolve with the needs of the study over time at each site. For example, membership could expand to include other individuals with lived experience as appropriate (e.g., partners of pregnant people with OUD, adult children of parents that used opioids during pregnancy). Of note, there is an ethical responsibility to ensure the make-up of the stakeholder group allows all individuals with lived experience to feel their voice is able to be heard. It may at times be appropriate to establish different types of advocacy boards.

In conclusion, these stakeholder meetings provided very valuable information for structuring upcoming studies; however, researchers would have benefitted from more time and more opportunities for in-person connection. Additionally, ongoing dialogue and relationship building with stakeholders is needed, particularly people with lived experience. Research and funding agencies must be flexible in timelines and methods to allow for incorporation of stakeholder input.

## Data Availability Statement

The original contributions presented in the study are included in the article/Supplementary Material, further inquiries can be directed to the corresponding author/s.

## Ethics Statement

The studies involving human participants were reviewed and approved by New York University Institutional Review Board. Written informed consent for participation was not required for this study in accordance with the national legislation and the institutional requirements.

## Author Contributions

JD contributed to the conception, design, analysis, interpretation, and drafting of the manuscript. KM contributed to the conception, design, interpretation, and drafting of the manuscript. EK contributed to the conception, design, and funding of the HBCD Phase I study and design, analysis, interpretation, and revising the manuscript. KS and RB contributed to the conceptualization, data acquisition, and revising the manuscript. KP contributed to data acquisition and manuscript editing. JG and TV contributed to data acquisition. AP, HG, and MT contributed to the conception, design, and funding of the HBCD Phase I study and data acquisition and manuscript editing. ES contributed to the design, data acquisition, and manuscript editing. AG contributed to the conception, design, and funding of the HBCD Phase I study and conception, design, analysis, interpretation, and drafting of the manuscript. All authors have approved the manuscript.

## Conflict of Interest

The authors declare that the research was conducted in the absence of any commercial or financial relationships that could be construed as a potential conflict of interest.

## Publisher's Note

All claims expressed in this article are solely those of the authors and do not necessarily represent those of their affiliated organizations, or those of the publisher, the editors and the reviewers. Any product that may be evaluated in this article, or claim that may be made by its manufacturer, is not guaranteed or endorsed by the publisher.
