# Supplementary material for: Stakeholder Perspectives on Advancing Understanding of Prenatal Opioid Exposure and Brain Development From the iOPEN Consortium of the Healthy Brain and Child Development Study
Source: Front Psychol. 2021 Jul 30;12:698766. doi: 10.3389/fpsyg.2021.698766 (PMC8361604; doi:10.3389/fpsyg.2021.698766)
Supplement: Supplementary file 1 [file Data_Sheet_1.docx]

**Appendix A.** Template for stakeholder meetings.

1. Who else should be here?
2. Potential benefits of a study of this kind for individuals, families and communities?
3. Ways to maximize potential benefits?
4. Potential ethical and legal considerations of a study of this kind?
